# A retrospective study on the efficacy and safety of bone cement in the treatment of endplate fractures

**DOI:** 10.3389/fsurg.2022.999406

**Published:** 2022-10-06

**Authors:** Zhijian Zhao, Lei Deng, Xi Hua, Haojun Liu, Hao Zhang, Xuejun Jia, Rushuai Wei, Mingming Liu, Nanning Lv

**Affiliations:** ^1^ Department of Orthopedic Surgery, The Second People's Hospital of Lianyungang, Lianyungang, China; ^2^ Department of Orthopaedics, The First Affiliated Hospital of Soochow University, Soochow University, Suzhou, China

**Keywords:** percutaneous kyphoplasty, endplate, bone cement, osteoporosis, fracture

## Abstract

**Background:**

Endplate fractures is an important factor affecting the curative effect of percutaneous kyphoplasty for spinal fracture. The purpose of this study is to investigate the effect of sealing endplate fracture with bone cement on minimally invasive treatment of spinal fracture.

**Methods:**

A total of 98 patients with osteoporotic vertebral fractures combined with endplate fractures treated with bone cement surgery in our hospital were retrospectively analyzed. They were grouped according to whether bone cement was involved in the endplate fractures. Group A: bone cement was not only distributed in the fractured vertebral body, but also dispersed into the endplate fractures. Group B: bone cement was confined to the fractured vertebra but did not diffuse into the cracks of the endplate. The basic information, imaging changes of the fractured vertebral body, VAS score, ODI score, bone cement distribution and postoperative complications of the two groups were analyzed and compared.

**Results:**

The height of the injured vertebra and the kyphotic Cobb angle in the two groups were significantly improved after surgery, but the anterior height of the vertebra in group B was lower than that in group A and the kyphotic Cobb angle was higher than that in group A at the last follow-up (*P *< 0.05). VAS score and ODI score in 2 groups were significantly improved after operation (*P *< 0.05), but the VAS score and ODI score in group A were lower than those in group B at the last follow-up (*P *< 0.05). The incidence of bone cement leakage and adjacent vertebral fracture in group A was higher than that in group B (*P *< 0.05).

**Conclusion:**

Diffusion of bone cement into the cracks of the endplate may also restore and maintain the height of the injured vertebra, relieve pain and restore lumbar function. However, diffusion of bone cement into the cracks of the endplate can increase the incidence of cement leakage and adjacent vertebral fractures.

## Introduction

Osteoporosis is a common orthopedic disease in the elderly, and osteoporotic vertebral compression fracture is one of the most common complications of osteoporosis (1, 2). With the acceleration of the aging process of China's population, the incidence of osteoporotic vertebral compression fractures has increased yearly, one of the incidences of women is higher than that of men (3). Osteoporotic vertebral fractures cause severe low back pain, decreased vertebral height and kyphosis, which seriously affect the life quality of patients (4). Bone cement-reinforced vertebral fractures are currently an important method for the clinical treatment of osteoporotic vertebral compression fractures, including percutaneous vertebroplasty (PVP) and percutaneous kyphoplasty (PKP), which can effectively relieve pain and stabilize fractured vertebral bodies in patients (5, 6). For patients with vertebral fractures, the formation of microscopic nooses between bone cement and trabecular bone and the elimination of fracture fretting are important factors to relieve pain and restore spinal biomechanics (7).

In the treatment of osteoporotic vertebral compression fractures, we often pay too much attention to the mechanical recovery of the fractured vertebral body, while the treatment of endplate fractures and adjacent disc injuries is often neglected. The endplate is the intermediary that connecting the vertebral body and intervertebral disc. The endplate transmits the load of the human body and plays an important role in undertaking the nutrient exchange and stress buffering of the intervertebral disc (8). For patients with endplate fractures, it is often difficult to disperse bone cement into the endplate cracks owing to the risk of bone cement leakage into the intervertebral space causing intervertebral disc damage. Is it better to pack endplate cracks with bone cement to stabilize the fracture fragment? Or is it better to limit the cement to the inside of the vertebral body to avoid cement leakage? At present, the clinical application of bone cement in endplate cracks is still unclear. This study retrospectively analyzed the patients with osteoporotic vertebral compression fractures with endplate cracks treated by percutaneous kyphoplasty. The influence of cement leakage and clinical efficacy was aimed to provide certain theoretical guidance for the targeted application of bone cement in the treatment of osteoporotic fractures.

## Methods

### Study design and participants

A total of 98 patients with osteoporotic vertebral fractures combined with endplate fractures who received bone cement surgery in our hospital from January 2017 to December 2020 were retrospectively analyzed. The relevant information of patients before and after surgery was collected. The work has been reported in line with the STROCSS criteria.

**Figure 1:**
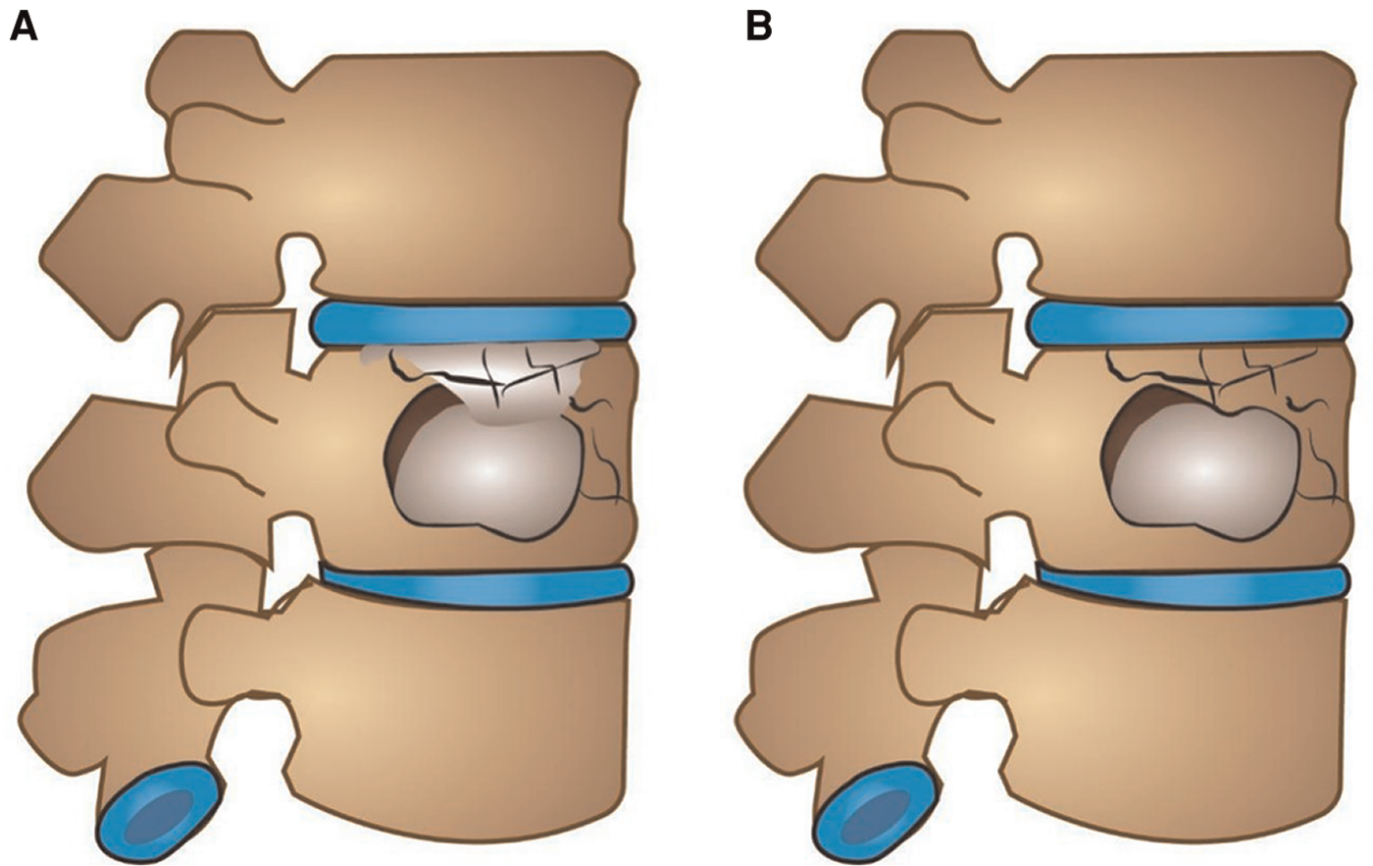
Schematic diagram of both groups. (**A**) The bone cement was not only distributed in the fractured vertebral body, but also diffused into the cracks of the endplate. (**B**) The bone cement was only confined in the fractured vertebral body but did not diffuse into the endplate cracks.

**Figure 2:**
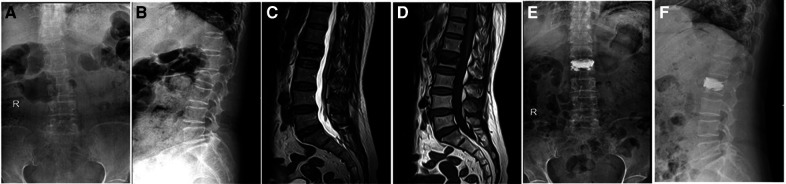
In group A, a patient with L2 vertebral fracture with upper endplate fracture was treated with bone cement, and the bone cement was completely filled in the fracture fissure of upper endplate. (**A–B**) Preoperative x-ray. (**C–D**) Preoperative MRI. (**E–F**) x-ray after surgery.

**Figure 3:**
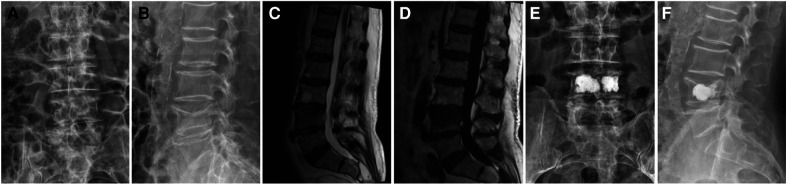
In group B, a patient with L4 vertebral fracture with upper endplate fracture was treated with bone cement, and no or a small amount of bone cement diffused into the fracture crack of the upper endplate. (**A–B**) Preoperative x-ray. (**C–D**) Preoperative MRI. (**E–F**) x-ray after surgery.

Inclusion criteria: fresh vertebral fractures diagnosed by preoperative x-ray, CT, and MRI (MRI was performed to observe intravertebral hemorrhage and bone marrow edema. Extensive hyperintensity and/or definite hyperintensity fracture line changes were observed on T2 lipid-suppression sequence.); combined with endplate fractures; fractured vertebral body with intact posterior wall of vertebral body without symptoms of spinal canal and nerve compression; single vertebral disease patients. Exclusion criteria: old fracture; primary or metastatic spinal tumor; spinal infection; abnormal coagulation function and mental abnormality; posterior vertebral body collapse defect accompanied by symptoms of dural sac or nerve tissue compression. Grouping was based on whether bone cement filled into endplate fractures (as shown in Figure 1). Group A: The bone cement was not only distributed in the fractured vertebral body, but also diffused into the cracks of the endplate, a total of 46 cases (Figure 2 shows the imaging data of a typical case). Group B: The bone cement was only confined in the fractured vertebral body but did not diffuse into the endplate cracks, a total of 52 cases (Figure 3 shows the imaging data of a typical case). All patients were followed up for at least 1 year.

### Surgical methods

The patient was treated with percutaneous kyphoplasty. The patient was placed in the prone position under local anesthesia. Accurately locate the fractured vertebral body under C-arm x-ray fluoroscopy. Confirm the lateral compression position of the vertebral body, and routinely disinfect the towel. Under fluoroscopy, a puncture needle was used to enter the pedicle through the skin puncture point on one or both sides of the injured vertebra. After adjusting the angle of the puncture needle, the needle was punctured to the anterior 1/3 of the vertebral body, and a working sleeve was placed. Insert a balloon dilator along the working channel, slowly pressurize the expansion balloon to restore the height of the vertebral body and release the pressure to withdraw the balloon. The bone cement was prepared and slowly injected into the vertebral body under the monitoring of the C-arm machine after waiting for it to become filamentous. The injection was stopped when the bone cement dispersed satisfactorily or the bone cement leaked. Intermittently rotate the cannula, pull out the cannula after the bone cement solidifies, and cover it with a sterile dressing.

### Assessed parameters

The imaging data, clinical efficacy and postoperative complications of all patients were analyzed 1 day before operation, 2 days after operation and 1 year after operation.

Imaging data: The frontal and lateral x-ray images of the injured vertebra were collected, respectively before and after the operation from all patients. The height of the anterior edge of the vertebral body and the kyphotic Cobb angle (The angle between the parallel lines between the superior endplate of the injured vertebra and the inferior endplate of the inferior vertebral body) were measured before and after the operation.

Clinical efficacy: Visual analogue scale (VAS) and Oswestry disability index (ODI) scores were recorded before and after surgery.

Adverse reactions: Complications such as bone cement leakage, adjacent vertebral fractures and refractures after surgery were recorded.

### Statistical analysis

SPSS 13.0 statistical software was used for data analysis, and the data were expressed in the form of mean ± standard error. The independent sample t-test was used for analysis for two groups comparison. Analysis of variance was used for multi-sample comparison. Differences were considered statistically significant at *P *< 0.05.

## Results

### General data

**Figure 4:**
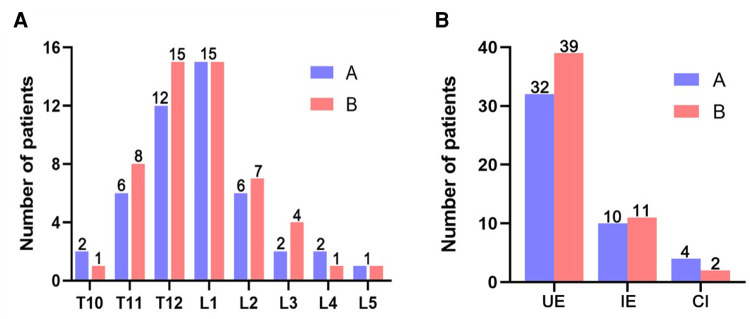
Injured vertebral segment and endplate injury site. (**A**) Comparison of vertebral injury segments between two groups. (**B**) Comparison of endplate injury site between two groups. UE: Only the upper endplate was damaged. IE: Only the Inferior endplate was damaged. CI: Both the upper endplate and the lower endplate were damaged.

The demographic data of both groups was shown in Table 1. A total of 98 patients were included in this study, including 46 patients in group A and 52 patients in group B. All patients were followed up for at least 1 year. There was no significant difference in age, sex, bone mineral density, preoperative VAS, and preoperative ODI between the two groups (*P *> 0.05), so these patients were comparable. There were no significant differences in bone cement injection volume and times of fluoroscopy between the two groups (*P *> 0.05). The fractured vertebral bodies of the two groups of patients are shown in Figure 4.

**Table 1:** Comparison of basic data and intraoperative related information.

	Group A (*n* = 46)	Group B (*n* = 52)	*P*-value (A vs. B)
Age (years)	64.85 ± 9.03	63.12 ± 8.83	0.342
Gender			
Male	12	13	0.279
Female	34	39	
Follow-up time (month)	18.13 ± 3.39	17.98 ± 3.07	0.819
Operation time (min)	22.93 ± 4.24	23.79 ± 4.05	0.311
Bone mineral density	−2.46 ± 0.45	−2.49 ± 0.5	0.708
x-ray time	23.59 ± 3.37	22.62 ± 3.31	0.154
Cement dosage (ml)	5.16 ± 1.04	4.85 ± 0.95	0.118
Pre-op. VAS	7.87 ± 0.98	8.06 ± 1.04	0.360
Pre-op. ODI	78.07 ± 6.74	79.42 ± 6.89	0.333
Pre-op. AHD (mm)	17.14 ± 2.25	17.55 ± 2.30	0.368
Pre-op. Cobb angles	21.07 ± 4.57	22.62 ± 4.31	0.087

AHD, Anterior height of diseased vertebrae.

### Imaging data

**Figure 5:**
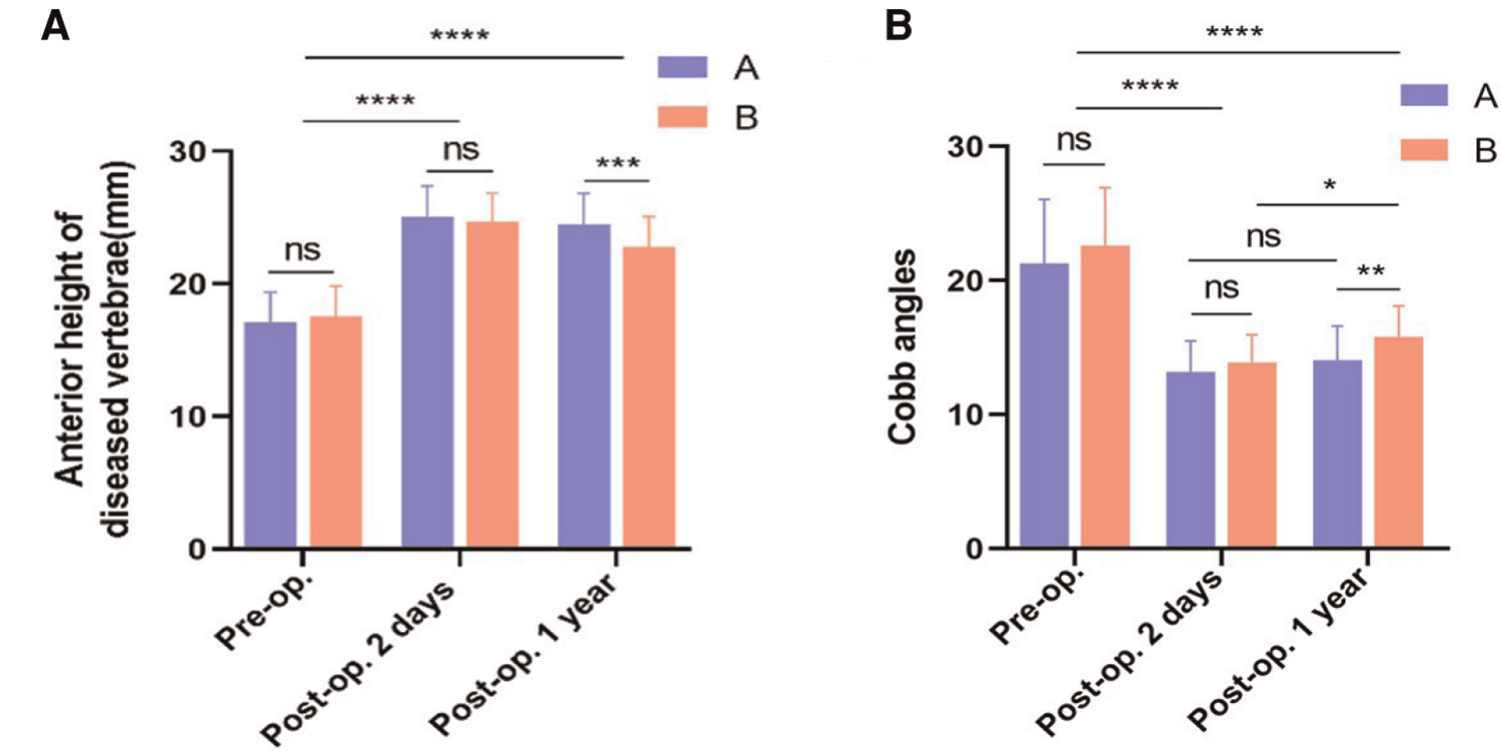
Vertebral imaging data. (**A**) Comparison of anterior height of diseased between two groups. (**B**) Comparison of Cobb angles between two groups. *, *P* < 0.05 vs. control; **, *P* < 0.001 vs. control; ***, *P* < 0.0001 vs. control.

The height of the anterior edge of the vertebral body of both groups was shown in Table 2. There was no significant difference in the height of the anterior edge of the vertebral body between the two groups before surgery (*P *> 0.05). There was no significant difference in the height of the anterior edge of the vertebral body between the two groups after surgery (*P *> 0.05), but the height of the anterior edge of the vertebral body in the group B was lower than that in the group A at the last follow-up, and the difference between the two groups was statistically significant (*P *< 0.05). (As shown in Figure 5).

**Table 2:** Anterior height of diseased vertebrae (mean ± SD; mm).

Classify	Group A (*n* = 46)	Group B (*n* = 52)	*P*-value (A vs. B)
Pre-op.	17.14 ± 2.25	17.55 ± 2.30	0.368
Post-op. 2 days	25.05 ± 2.34	24.69 ± 2.18	0.431
Post-op. 1 year	24.50 ± 2.34	22.82 ± 2.26	<0.001
*P*. (pre. vs. 2 d.)	<0.0001	<0.0001	–
*P*. (2 d. vs. 1 y.)	0.264	<0.001	–

Pre-op., pre-operation; Post-op., Post-operation; 2 d., 2 days; 1 y., 1 year; *P*., *P*-value.

The Cobb angle of kyphosis of both groups was shown in Table 3. There was no significant difference in the Cobb angle of kyphosis between two groups before surgery (*P *> 0.05), and the Cobb angle of kyphosis between the two groups was significantly improved after surgery and at the last follow-up (*P *< 0.05). There was no significant difference in the Cobb angle of kyphosis between the two groups after operation (*P *> 0.05), but the Cobb angle of kyphosis in group B was greater than that in group A at the last follow-up, and the difference between the two groups was statistically significant (*P *< 0.05). (As shown in Figure 5).

**Table 3:** Cobb angles (mean ± SD; °).

Classify	Group A (*n* = 46)	Group B (*n* = 52)	*P*-value (A vs. B)
Pre-op.	21.07 ± 4.57	22.62 ± 4.31	0.087
Post-op. 2 days	13.05 ± 2.26	13.89 ± 2.07	0.057
Post-op. 1 year	13.82 ± 2.50	15.81 ± 2.30	0.014
*P*. (pre. vs. 2 d.)	<0.0001	<0.0001	–
*P*. (2 d. vs. 1 y.)	0.500	0.005	–

Pre-op., pre-operation; Post-op., Post-operation; 2 d., 2 days; 1 y., 1 year; *P*., *P*-value.

### Clinical efficacy

**Figure 6:**
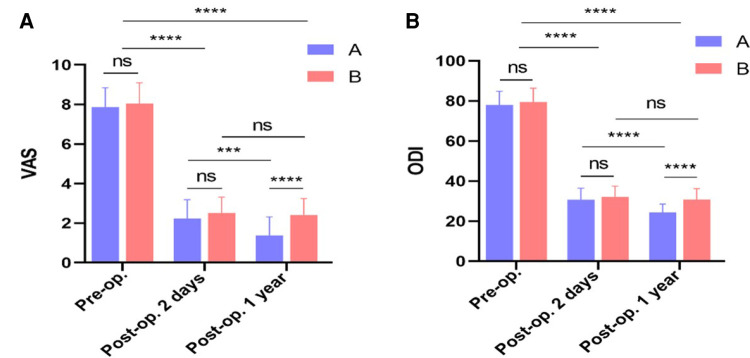
Clinical efficacy. (**A**) Comparison of VAS between two groups. (**B**) Comparison of ODI between two groups. ***, *P* < 0.001 vs. control; ****, *P* < 0.0001 vs. control.

The VAS and ODI of both groups were shown in Tables 4, 5. There was no significant difference in the preoperative VAS score and ODI score between two groups (*P *> 0.05). After operation, the VAS score and ODI score of the two groups were significantly improved (*P *< 0.05), but there was no significant difference (*P *> 0.05) between the two groups. The last follow-up found that the VAS score and ODI score of group A were lower than those of group B, and the difference was statistically significant (*P *< 0.05). (As shown in Figure 6).

**Table 4:** Visual analogue scale (VAS; mean ± SD).

Classify	Group A (*n* = 46)	Group B (*n* = 52)	*P*-value (A vs. B)
Pre-op.	7.87 ± 0.98	8.06 ± 1.04	0.360
Post-op. 2 days	2.24 ± 0.95	2.52 ± 0.80	0.117
Post-op. 1 year	1.39 ± 0.93	2.42 ± 0.82	<0.0001
*P*. (pre. vs. 2 d.)	<0.0001	<0.0001	–
*P.* (2 d. vs. 1 y.)	<0.001	0.848	–

Pre-op., pre-operation; Post-op., Post-operation; 2 d., 2 days; 1 y., 1 year; *P*., *P*-value.

**Table 5:** Oswestry disability index (ODI; mean ± SD).

Classify	Group A (*n* = 46)	Group B (*n* = 52)	*P*-value (A vs. B)
Pre-op.	78.07 ± 6.74	79.42 ± 6.89	0.333
Post-op. 2 days	31.37 ± 5.66	32.12 ± 5.25	0.213
Post-op. 1 year	24.35 ± 4.11	30.77 ± 5.37	<0.0001
*P*. (pre. vs. 2 d.)	<0.0001	<0.0001	–
*P*. (2 d. vs. 1 y.)	<0.0001	0.482	–

Pre-op., pre-operation; Post-op., Post-operation; 2 d., 2 days; 1 y., 1 year; *P*., *P*-value.

### Postoperative complications

**Figure 7:**
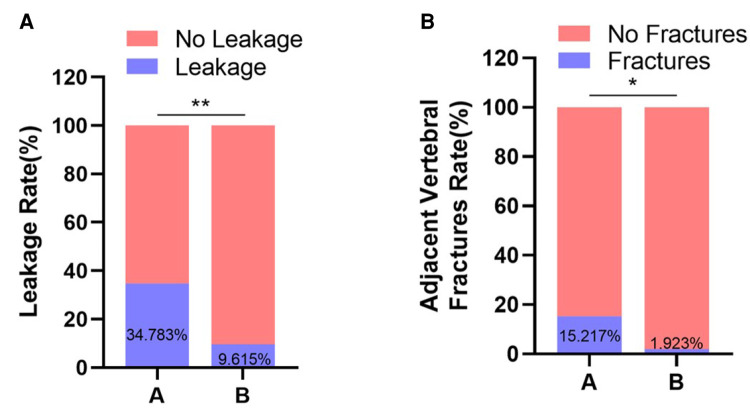
Postoperative complications. (**A**) Comparison of bone cement leakage rate between two groups. (**B**) Comparison of adjacent vertebral fractures between two groups. *, *P* < 0.05 vs. control; **, *P* < 0.01 vs. control.

There were 16 cases of bone cement leakage in group A, among which 12 cases were intervertebral disc leakage and 4 cases were paravertebral. There were 5 cases of bone cement leakage in group B, including 2 cases of anterior vertebral leakage, 1 case of intervertebral disc, and 2 cases of paravertebral body leakage. The bone cement leakage rate in group A was higher than group B (*P *< 0.05) as shown in Figure 7. During the postoperative follow-up, 7 patients in group A had adjacent vertebral fractures, and 1 in group B. There was statistically significant difference in the incidence of adjacent vertebral fractures between the two groups (*P *< 0.05) (as shown in Figure 7). Further, we calculate the number needed to harm (NNH) on 7.52 vertebral bodies are treated with bone cement at the endplate fractures to “produce” one postoperative complication.

## Discussion

The endplate is the intermediary that connects the vertebral body and the intervertebral disc and transmits the load of the human body. Each endplate includes two parts: the bone endplate and the cartilage endplate. The former is the structure covered by cartilage in the vertebral body, and the latter is responsible for the nutrient exchange and stress of the intervertebral disc (9). Endplate rupture injury mostly occurs in the center or anterior part of the endplate. When it is severely ruptured, the nucleus pulposus will lose function and enter the vertebral body (10). Endplate and annulus fibrosus are rich in innervation of nerve endings, and their damage is an important cause of low back pain with a high incidence in OVCF (11). The complex of endplate and intervertebral disc is unstable. During spinal extension and flexion activities and weight-bearing walking, nerve endings in the injured area are stimulated to cause pain, and this injury will not heal for a long time due to continuous breathing movement of the thorax and spinal movement. A common cause of chronic back pain that persists over time (12).

Since the application of bone cement filling technology in spine surgery, minimally invasive surgery represented by PVP and PKP has been widely used in diseases such as osteoporotic vertebral compression fractures and achieved good results (13, 14). However, it cannot be ignored that the incidence of postoperative complications is relatively high. The long-term loss of vertebral height and the aggravation of kyphosis after surgery lead to chronic pain and limited mobility in patients. The treatment of such complications is quite difficult (15). Previous studies have shown that osteoporotic vertebral compression fractures are often accompanied by vertebral endplate fractures, and endplate fractures are one of the main risk factors for vertebral height loss after thoracolumbar fractures (16). From the perspective of biomechanical research, the vertebral body endplates bear 40%–75% of the vertebral body pressure are directly involved in the transfer of pressure from the intervertebral disc to the vertebral body. Even a slight change in the shape of the endplate will lead to significant vertebral body motor function (17). However, it is often difficult to completely correct the deformity of the endplate during surgery, which leads to increased stress in the perivertebral portion of patients with endplate fractures. In addition, the aggravation of kyphosis is related to the insertion of the intervertebral disc into the vertebral body or endplate from the fracture of the endplate, and the intervertebral disc embedded in the vertebral body or endplate is more likely to lose the height of the injured vertebral body due to necrosis after surgery (18). Therefore, the loss of postoperative vertebral height and the occurrence of kyphosis are closely related to the biomechanical changes of the vertebral body caused by changes in the stress distribution of the endplates (19). This study also confirmed that patients who did not fill endplate fractures with bone cement had postoperative vertebral height reduction and increased kyphosis. However, in patients with bone cement diffused to the endplate fractures, there was no significant change in vertebral body height and kyphosis after surgery. This indicated that the re-collapse of the fractured vertebral body could be prevented to a certain extent by sealing the fracture of the endplate with bone cement. On the other hand, the study also found that the VAS score and ODI score at last follow-up of patients with unsealed endplate fractures were also higher than those of patients with endplate fractures sealed with bone cement, which may be due to the endplate fracture line insufficient diffusion of the bone cement at the site and difficulty in maintaining the stability of the bone around the fracture line led to fretting of the endplate fracture. In addition, the long-term pain exacerbation in patients with unsealed endplate fractures with bone cement was also closely related to the decrease in the height of the injured vertebra and the exacerbation of kyphosis.

Studies have shown that endplate fractures increase the risk of bone cement leakage into the intervertebral disc, which is also an important cause of low back pain after fracture surgery and a high-risk factor for refracture of adjacent vertebral bodies later (20). Biomechanical studies have shown that bone cement-reinforced vertebrae conduct excessive stress through the intervertebral disc to adjacent vertebral bodies, which may lead to refractures of adjacent vertebral bodies (21). After the bone cement leaks into the intervertebral disc, the distance between the cement and the endplate of the adjacent vertebra is closer, and the bone cement leaking into the intervertebral disc produces a concentrated stress effect on the adjacent vertebra. The effect of small shock absorption, thereby increasing the stress transmission of the strengthened vertebra to the adjacent vertebral body, has become an important risk factor for refracture of the adjacent vertebral body (22). This study also found that the bone cement sealing of endplate fractures increased the risk of bone cement intervertebral disc leakage, and the incidence of postoperative refracture was also higher than that of the non-cemented endplate fracture group.

In conclusion, for OVCF patients with endplate fractures, the closure of endplate cracks with bone cement can effectively strengthen the fractured vertebral body, maintain the postoperative vertebral height, relieve pain, and restore lumbar vertebral function well. However, the incidence of postoperative bone cement leakage and refracture of adjacent vertebral bodies is high. Therefore, when using bone cement to strengthen the fractured vertebral body in the treatment of OVCF patients with endplate fractures, the purpose of surgery should include sealing and repairing endplate fissures, preventing leakage of bone cement into the intervertebral space, and preventing intervertebral disc herniation in addition to supporting the fractured vertebral body and endplate gap. The surgical strategy is to choose PKP as much as possible, reducing the collapsed endplate through balloon dilation, creating a cavity in the vertebral body, forming a dense bone to seal the fracture fissure, and adjusting the bone cement to be more viscous and reduce the bolus pressure to reduce the cement to the wall. External (disc) leaks are possible, and injections should be discontinued as soon as leakage occurs. In addition, more attention should be paid to comprehensive measures such as long-term anti-osteoporosis treatment, functional exercise of lumbar back muscles, analgesia, physiotherapy, and psychological treatment after operation. However, there are still shortcomings in this study. Because it is a retrospective study, it cannot fully demonstrate the impact of endplate fractures, intervertebral disc injuries and other factors on the surgical effect. The realization of the diffuse distribution pattern of bone cement in the diseased vertebra are also needed to be further explored.

## Conclusion

Diffusion of bone cement into the cracks of the endplate may also restore and maintain the height of the injured vertebra, relieve pain and restore lumbar function. However, diffusion of bone cement into the cracks of the endplate can increase the incidence of cement leakage and adjacent vertebral fractures.

## Data Availability

The original contributions presented in the study are included in the article/Supplementary Material, further inquiries can be directed to the corresponding author/s.
